# Acupuncture points injection mitigates chronic pain through transient receptor potential V1 in mice

**DOI:** 10.22038/IJBMS.2022.60121.13327

**Published:** 2022-04

**Authors:** Hsien-Yin Liao, Ming-Chia Lin, Yi-Wen Lin

**Affiliations:** 1 College of Chinese Medicine, School of Post-Baccalaureate Chinese Medicine, China Medical University, Taichung 40402, Taiwan; 2 Department of Nuclear Medicine, E-DA Hospital, College of Medicine, I-Shou University, Kaohsiung 82445, Taiwan; 3 College of Chinese Medicine, Graduate Institute of Acupuncture Science, China Medical University, Taichung 40402, Taiwan; 4 Chinese Medicine Research Center, China Medical University, Taichung 40402, Taiwan

**Keywords:** Acupuncture points – injection, Chronic inflammatory – pain, Dorsal root ganglion, Iba1, Somatosensory cortex, TRPV1

## Abstract

**Objective(s):**

Tissue injury in peripheral sites can result in long-term potentiation in nociceptive neurons and surrounding glial cells, potentially resulting in the development of chronic inflammatory pain (CIP). Acupoint injection (AI) is similar to Western phototherapy, which injects solutions at specific sites to mitigate chronic pain. AI has shown greater benefits compared with acupuncture. In this study, we examined the therapeutic effect and explored the underlying mechanisms of AI in mice CIP model.

**Materials and Methods:**

We injected thrice complete Freund’s adjuvant (CFA) into the mouse’s hind paw to induce CIP.

**Results:**

We found that, after two weeks, CFA injection significantly induced mechanical and thermal hyperalgesia which were attenuated by AI treatment. Transient receptor potential V1 (TRPV1) channels and associated molecules were all increased in CIP in mice dorsal root ganglion (DRG), spinal cord (SC), thalamus, and somatosensory cortex (SSC). The aforementioned molecules were mitigated in AI and Trpv1 knockout mice. Furthermore, Iba1-positive cells (microglial marker) were also potentiated and shared a similar tendency with TRPV1.

**Conclusion:**

These findings suggest that AI can alleviate chronic pain by reducing TRPV1 overexpression in both neuronal and microglial cells. Our results suggest new potential therapeutic targets for AI in chronic pain.

## Introduction

Acupoint injection (AI) has increased in popularity as a complementary therapy for chronic pain. The most common form involves injection of a glucose solution to induce local inflammation and initiate a wound-healing cascade. AI is often utilized to relieve the pain of ligaments, tendons, and articular cartilage. After injection of concentrated glucose, the damaged tissue is repaired to form a denser, stronger, and tighter ligament or tendon (1, 2). In the clinic, AI is usually performed 4–6 courses to ensure tissue repair and pain relief (3, 4). Several other solutions have also been used for AI, including dextrose and phenol. Although AI has demonstrated efficacy for healing local tissue damage, the underlying mechanisms of action are still unclear. One possibility is that dextrose induces osmotic damage to local neurons, which in turn attracts brain-resident inflammatory mediators to initiate the wound-healing cascade (5). Local osmotic damage may also induce infiltration of granulocytes, followed by monocytes and macrophages (6). However, elucidation of the actual mechanisms requires the use of appropriate animal models.

Another potential mechanism involves the transient receptor potential vanilloid 1 (TRPV1) channel responsive to thermal, mechanical, and capsaicin stimulation and expressed by peripheral sensory neurons. Activation of TRPV1 channels triggers cation influx, including Ca^2+^, which activates downstream signaling pathways causing peripheral and central pain sensitization (7, 8). Peripheral inflammation induced by painful (potentially tissue-damaging) stimulation and local injury may also enhance signal propagation from the periphery to the somatosensory cortex via the dorsal root ganglion, spinal cord, and thalamus, termed central sensitization. This may lead to allodynia, a condition in which even light touch and other normally non-painful stimuli are perceived as painful (7, 8). The TRPV1 channel and related downstream signaling molecules are involved in both acute pain and central sensitization, and TRPV1 was recently shown to detect neuroinflammation in mice (9, 10). Activation of TRPV1 further increases the mitogen-activated protein kinase (MAPK) family involved in the pain pathway. MAPK is composed of extracellular signal-regulated protein kinase (ERK), p38, and c-Jun N-terminal kinase/stress-activated protein kinase (JNK) (11). These kinases have been reported in neuronal plasticity, central sensitization, and cognitive function (12). The pPI3K-pAkt-pmTOR pathway is well known in nociception of central sensitization (13).

Acupuncture has been used for over 3,000 years to treat pain and associated disorders. Modern acupuncture techniques have proven to be convenient, safe, and inexpensive approaches for treating diseases (14-15). These therapies are particularly beneficial for lower back pain, inflammatory pain, and muscle pain due to exercise, fibromyalgia (FM), and myofascial pain (16-20). Recent studies have implicated release of adenosine (21), dopamine (22), and endogenous opiates (23) in the analgesic effects of acupuncture as evidenced by elevated levels of these molecules in serum or cerebrospinal fluid.

In the current study, we examined the efficacy of AI against chronic inflammatory pain (CIP) and determined the underlying mechanisms for its effect. We hypothesized that AI of high concentration glucose would be effective for treating chronic mechanical and thermal hyperalgesia in mice by regulating TRPV1 signaling from the periphery to the somatosensory cortex.

## Materials and Methods

***Animals***

We used 8–12 week old C57BL/6 mice for all experiments, which were purchased from BioLASCO Co. Ltd. (Taipei, Taiwan). The mice were randomly assigned to four groups (n = 8 per group): (1) Normal, (2) CIP, (3) AI, and (4) *Trpv1*^-/-^. The sample size required for an alpha of 0.05 and a power of 80% was eight animals per group. After arrival, the mice were housed in a room under a 12/12 hr light/dark cycle with water and food available *ad libitum*. All procedures were approved by the Institutional Animal Care and Use Committee of China Medical University (No. 2018-110) and were conducted in accordance with the Guide for the Use of Laboratory Animals provided by the National Research Council and the ethical guidelines of the International Association for the Study of Pain. The number of animals used and their suffering were minimized.

***CIP induction***

Mice were randomly divided into four groups and then anesthetized with 1% isoflurane for CFA injections and AI treatment. Mice were next injected with 20 μl saline (pH 7.4, buffered with 20mM HEPES) or CFA 20 μl (complete Freund’s adjuvant; 0.5 mg/ml heat-killed M. tuberculosis (Sigma, St. Louis, MO) suspended in oil:saline 1:1 emulsion) in the plantar surface of the hind paw. CFA was used to induce intraplantar chronic inflammation. CFA was administered twice: at baseline and day 7. Four groups and their treatments were as follows: (1) normal group: normal saline injection; (2) CIP group: CFA injections to induce CIP; (3) AI group: CIP mice treated with AI; and (4) *Trpv1*^-/-^ group: CFA injections in *Trpv1*^-/-^ mice. We followed the methods of H-Y Huang *et al*. (17).

***Nociceptive behavioral examination***

The mechanical and thermal pain behaviors were determined 3 times on days 0, 7, and 14 throughout the experiment after induction of the CIP model. All mice were moved to the behavior analysis room and were adapted to the environment for at least 30 min before behavior tests. All experiments were performed at room temperature and the stimuli were applied only when the animals were calm but not sleeping or grooming. First, the von Frey filament test was conducted. Mechanical sensitivity was measured by testing the force of responses to stimulation with 3 applications of the electronic, calibrated von Frey filament (IITC Life Science Inc., USA). Mice were placed onto a metal mesh (75x25x45 cm) and covered with a plexiglass cage (10x6x11 cm). Subjects were then mechanically stimulated by the tip of the filament at the plantar region of the right hind paw. The filament gram counts were recorded when the stimulation caused the subject to withdraw its hind paw. Second, Hargreaves’ assessment was used to measure thermal pain behavior by testing the time of response to thermal stimulation with 3 applications using Hargreaves’ test IITC analgesiometer (IITC Life Sciences, SERIES8, Model 390G). The mice were placed in a plexiglass cage on top of a glass sheet. The thermal stimulator was positioned under the glass sheet and the focus of the projection bulb was aimed exactly at the middle of the plantar surface of the right hind paw. A cut-off time of 20 sec was set to prevent tissue damage. In the thermal paw withdrawal test, the nociception threshold was assessed using the latency of paw withdrawal upon stimulus and was recorded when the constant applied heat stimulation caused the subject to withdraw its hind paw. We followed the methods of H-Y Huang *et al*. (17).

***Acupuncture point injection***

AI was conducted in the morning (9:00–10:00 am) immediately after induction of CIP. A high concentration of glucose (20%, 20 µl) was injected at the bilateral side of ST36 at baseline and day 7. Similar to humans, the ST36 point is located longitudinally at 3 cun below the knee joint and intersects with the middle of the tibialis anterior muscle. Mice were placed into a fixation machine under anesthesia with 5% isoflurane for induction, which was then decreased to 1% for maintenance. Bilateral ST36 acupoints were selected, sterilized with 70% alcohol and iodine solution, and the glucose solution was injected at the 3–5 mm depth.

***Tissue sampling and western blot analysis***

The lumbar dorsal root ganglion (DRG), spinal cord (SC), full thalamus, and somatosensory cortex (SSC) neurons were excised immediately to extract proteins. Total proteins were prepared by homogenizing the tissues in lysis buffer containing 50 mM Tris-HCl (pH 7.4), 250 mM NaCl, 1% NP-40, 5 mM EDTA, 50 mM NaF, 1 mM Na_3_VO_4_, 0.02% NaNO_3_, and 1× protease inhibitor cocktail (Amresco, Solon, OH, USA). The extracted proteins (30 μg per sample according to the BCA protein assay) were subjected to 8% sodium dodecyl sulfate-Tris glycine gel electrophoresis and transferred to a PVDF membrane. The membrane was blocked with 5% non-fat milk in TBS-T buffer (10 mM Tris pH 7.5, 100 mM NaCl, 0.1% Tween 20), incubated with primary antibodies (1:1000, Alomone, Jerusalem, Israel): anti-tubulin, anti-TRPV1, anti-Nav1.7, anti-Nav1.8, anti-RAGE, anti-pmTOR, anti-pPI3K, anti-pAkt, anti-pERK, anti-pS100B, anti- pNFκB, anti-pCREB, anti-pPKC or anti-pPKA in TBS-T and 1% bovine serum albumin, and incubated for 1 hr at room temperature. A peroxidase-conjugated anti-rabbit antibody (1:5,000) was used as the secondary antibody. The bands were visualized using an enhanced chemiluminescent substrate kit (Pierce, Rockford, IL, USA) with LAS-3000 Fujifilm (Fuji Photo Film Co. Ltd., Tokyo, Japan). If appropriate, the image intensities of specific bands were quantified with NIH ImageJ software (Bethesda, MD, USA). The protein ratios were determined by dividing the target protein intensities by the intensity of α-tubulin or β-actin (internal control) in the same sample. The calculated ratios were adjusted by dividing the ratios from the same comparative group relative to the control. We followed the methods of H-Y Liao *et al*. 

***Immunofluorescence***

Mice were euthanized with a 5% isoflurane via inhalation and intracardially perfused with normal saline followed by 4% paraformaldehyde. The brain was immediately dissected and post-fixed with 4% paraformaldehyde at 4 °C for 3 days. The tissues were placed in 30% sucrose for cryoprotection overnight at 4 °C. The brain was embedded in an Optimal cutting temperature (OCT) compound and rapidly frozen using liquid nitrogen before storing the tissues at -80 °C. Frozen segments were cut at 20-μm width on a cryostat and then instantaneously placed on glass slides. The samples were fixed with 4% paraformaldehyde and then incubated with a blocking solution consisting of 3% BSA, 0.1% Triton X-100, and 0.02% sodium azide, for 1 hr at room temperature. After blocking, the samples were incubated with the primary antibody (1:200, Alomone), TRPV1, and Iba1, prepared in 1% bovine serum albumin solution at 4 °C, overnight. The samples were then incubated with the secondary antibody (1:500), 488-conjugated AffiniPure donkey anti-rabbit IgG (H + L), 594-conjugated AffiniPure donkey anti-goat IgG (H + L), and Peroxidase-conjugated AffiniPure donkey anti-mouse IgG (H + L) for 2 hr at room temperature before being fixed with coverslips for immunofluorescence visualization. The samples were observed by an epi-fluorescent microscope (Olympus, BX-51, Japan) with a 20 x numerical aperture (NA = 1.4) objective. The images were analyzed by NIH Image J software (Bethesda, MD, USA). We followed the methods of H-Y Liao *et al*. 

***Statistical analysis***

Statistical analysis was performed using the SPSS statistics program. All of the data were expressed as the mean ± standard error (SEM). The Shapiro-Wilk test was performed to test the normality of data. Significant differences among all groups were tested using the repeated measure ANOVA, followed by a *post hoc* Tukey’s test. *P*<0.05 was considered significantly different.

## Results

***AI treatment suppresses TRPV1-dependent mechanical and thermal hyperalgesia***

**Figure 1:**
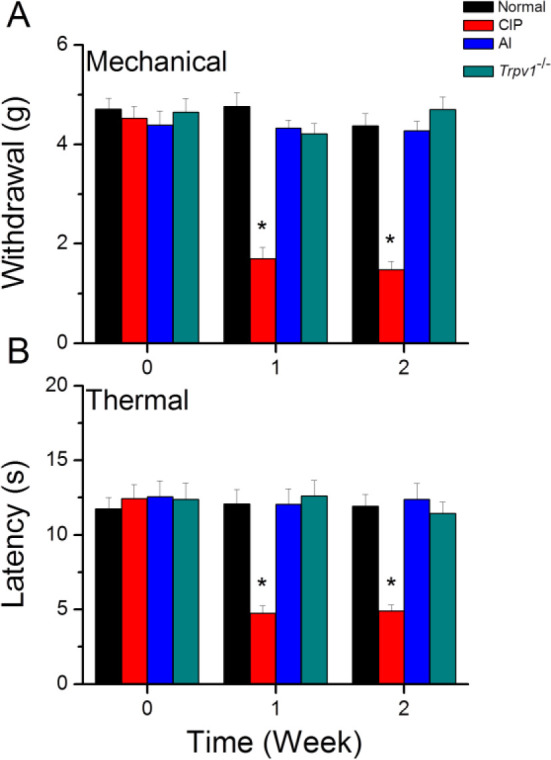
Mechanical withdrawal threshold and thermal latency in four groups of mice

To study the effect of EA in attenuating inflammatory pain, we compared responses of the von Frey filament and Hargreaves’ test among all groups. At baseline, all groups had similar mechanical responses that indicated no statistical significance among them. After injection with CFA to induce CIP, and following either control treatment or AI. One week after CIP induction, however, mechanical sensitivity was significantly elevated in the CIP group compared with the normal group, indicating hyperalgesia (Figure 1A, red column, n = 8). This mechanical hyperalgesia was significantly reversed by AI treatment (CIP + AI group) (Figure 1A, blue column, n = 8 mice). Similar to AI group mice, *Trpv1*^−/−^ mice (Figure 1A, green column, n = 8 mice) also exhibited little mechanical hyperalgesia following CIP.

The data concerning thermal hyperalgesia, measured by the Hargreave’s test, were similar to the data of mechanical hyperalgesia (Figure 1B). Thermal hyperalgesia was similar in all groups at basal conditions (Figure 1B, *P*>0.05). A significantly lower thermal pain latency was observed in the CIP group (Figure 1B, *P<0.05, red column). EA consistently reduced thermal hyperalgesia (Figure 1B, #*P*<0.05, blue column). Thermal hyperalgesia was also not detected in the TRPV1^−/−^ group (Figure 1B, #*P*<0.05, green column).

***Effects of CIP and AI on the expression levels of TRPV1 and associated signaling molecules in mouse dorsal root ganglia and spinal cord***

**Figure 2:**
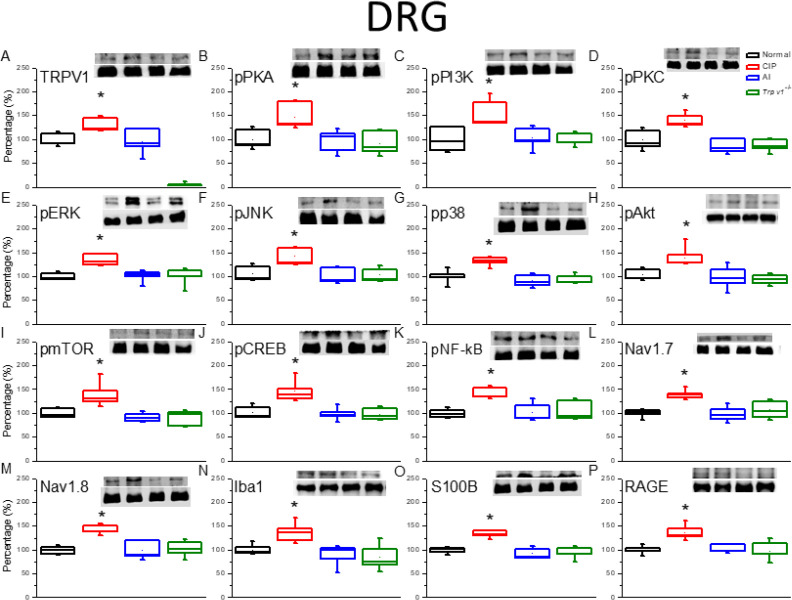
Expression levels of transient receptor potential V1 (TRPV1) and associated molecules in the mice dorsal root ganglion (DRG). The immunoblotting images depict four lanes of protein in the following order: Normal, chronic inflammatory pain (CIP), AI, and *Trpv1*^-/-^ groups. There are significant increases in protein expression in the CIP groups of (A) TRPV1, (B) phosphorylated (activated) PKA (pPKA), (C) pPI3K, (D) pPKC, (E) pERK, (F) pJNK, (G) pp38, (H) pAkt, (I) pmTOR, (J) pCREB, (K) pNFƙB, (L) Nav1.7, (M) Nav1.8, (N) Iba1, (O) S100B, and (P) RAGE levels, which were significantly attenuated in the AI and Trpv1-/- groups, depicting no difference when compared with the Normal group. * *P*<0.05 means a statistical difference compared with the normal group. The western blot bands at the top show the target protein. The lower bands are internal controls (β-actin or α-tubulin)

**Figure 3:**
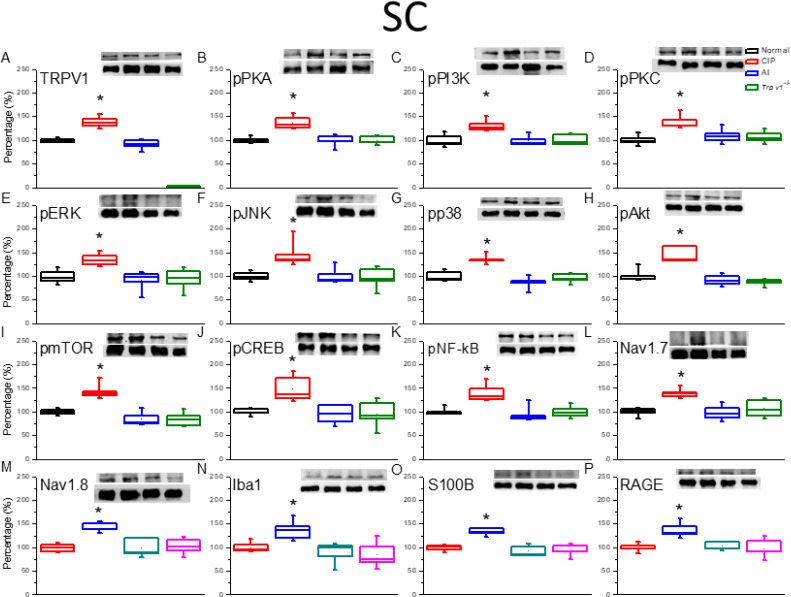
The expression levels of transient receptor potential V1 (TRPV1) and associated molecules in the mice spinal cord (SC). The immunoblotting images depict four lanes of protein in the following order: Normal, CIP, AI, *Trpv1*^-/-^ groups. There are significant increases in protein expression in the chronic inflammatory pain (CIP) groups of (A) TRPV1, (B) phosphorylated (activated) PKA (pPKA), (C) pPI3K, (D) pPKC, (E) pERK, (F) pJNK, (G) pp38, (H) pAkt, (I) pmTOR, (J) pCREB, (K) pNFkB, (L) Nav1.7, (M) Nav1.8, (N) Iba1, (O) S100B, and (P) RAGE levels, which were significantly attenuated in the AI and Trpv1-/- groups, depicting no difference when compared with the Normal group. * P<0.05 means a statistical difference compared with the normal group. The western blot bands at the top show the target protein. The lower bands are internal controls (β-actin or α-tubulin)

To examine the involvement of TRPV1 channels and related signaling factors in hyperalgesia and AI-mediated analgesia, changes in expression were examined by western blotting of DRG and SC tissue samples isolated after successful induction of hyperalgesia by CIP after reversal by AI (as confirmed by von Fey and Hargreaves’ tests). In the DRG (Figure 2), TRPV1 protein expression was significantly increased by CIP induction compared with sham-treated controls, and this augmentation was significantly reversed by AI. In addition, expression levels of phosphorylated (activated) PKA (pPKA) (Figure 2B), pPI3K (Figure 2C), and pPKC (Figure 2D) were significantly elevated in the DRG after CIP, and these responses were attenuated by AI. Increased expression levels were also attenuated following CIP in *Trpv1*^−/−^ mice compared with wild-type (WT) mice (CIP group). In addition, DRG expression levels of the phospho-activated mitogen-activated protein kinases (MAPKs) pERK (Figure 2E), pJNK (Figure 2F), and pp38 (Figure 2G) were elevated in CIP model mice, and these effects were reversed by AI and lower in *Trpv*1^−/−^ mice than WT mice following CIP induction. Expression levels of pAkt (Figure 2H) and pmTOR Figure 2I), two downstream effectors of pPI3K, and the transcription factors pCREB (Figure 2J) and NFκB (Figure 2K) were up-regulated during CIP, reversed by AI, and lower following CIP in *Trpv*1^-/-^ mice than WT mice. The nociception-associated Na channel subunits Nav1.7 (Figure 2L) and Nav1.8 (Figure 2M) demonstrated similar patterns of expression change. Finally, the activated microglial markers Iba1 (Figure 2N), S100B (Figure 2O), and RAGE (Figure 2P) were also elevated by CIP, and these responses were reversed by AI and lower in CIP model *Trpv*1^−/−^ mice than CIP model WT mice. Similar changes in protein expression were also observed in tissue samples from the SC (Figure 3).

***Increased TRPV1 and associated protein levels following CIP induction, reversal by AI, and suppression by Trpv1 gene deletion in the thalamus and somatosensory cortex***

**Figure 4:**
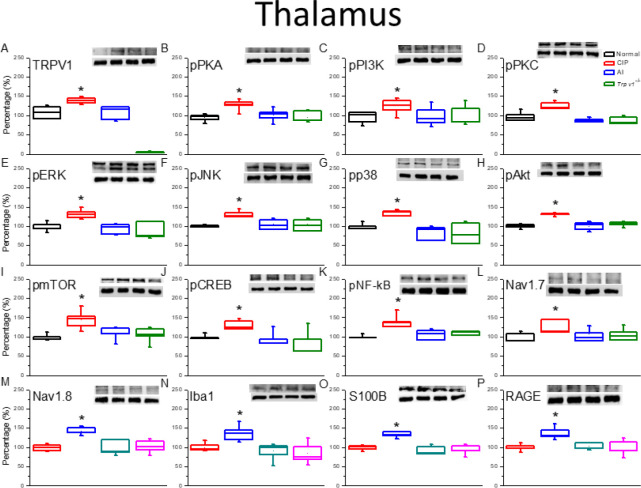
Expression levels of Transient receptor potential V1 (TRPV1) and associated molecules in the mice thalamus. The immunoblotting images depict four lanes of protein in the following order: Normal, CIP, AI, and Trpv1-/- groups. There are significant increases in protein expression in the CIP groups of (A) TRPV1, (B) pPKA, (C) pPI3K, (D) pPKC, (E) pERK, (F) pJNK, (G) pp38, (H) pAkt, (I) pmTOR, (J) pCREB, (K) pNFƙB, (L) Nav1.7, (M) Nav1.8, (N) Iba1, (O) S100B, and (P) RAGE levels, which were significantly attenuated in the AI and Trpv1-/- groups, depicting no difference when compared with the Normal group. * *P*<0.05 means a statistical difference compared with the normal group. The western blot bands at the top show the target protein. The lower bands are internal controls (β-actin or α-tubulin)

**Figure 5:**
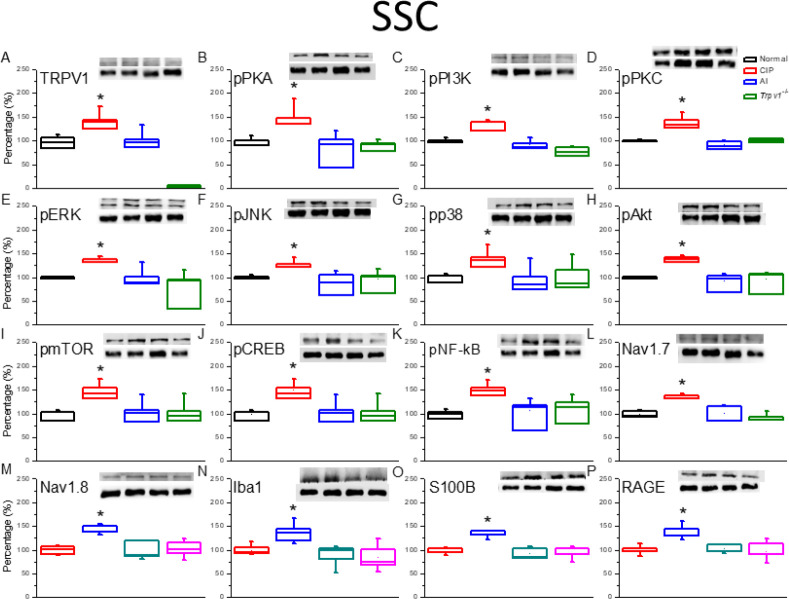
The expression levels of transient receptor potential V1 (TRPV1) and associated molecules in the mice somatosensory cortex (SSC). The immunoblotting images depict four lanes of protein in the following order: Normal, CIP, AI, and *Trpv1*^-/-^ groups. There are significant increases in protein expression in the chronic inflammatory pain (CIP) groups of (A) TRPV1, (B) phosphorylated (activated) PKA (pPKA), (C) pPI3K, (D) pPKC, (E) pERK, (F) pJNK, (G) pp38, (H) pAkt, (I) pmTOR, (J) pCREB, (K) pNFƙB, (L) Nav1.7, (M) Nav1.8, (N) Iba1, (O) S100B, and (P) RAGE levels, which were significantly attenuated in the AI and Trpv1-/- groups, depicting no difference when compared with the Normal group. * *P*<0.05 means a statistical difference compared with the normal group. The western blot bands at the top show the target protein. The lower bands are internal controls (β-actin or α-tubulin)

Samples of thalamus (Figure 4) and somatosensory cortex (Figure 5) were also examined by western blotting for changes in the expression levels of TRPV1 and downstream signaling molecules following CIP induction and AI. In accordance with findings in DRG and SC, there were significant increases in the expression levels of TRPV1 (Figures 4A, 5A), pPKA (Figures 4B, 5B), pPI3K (Figures 4C, 5C), pPKC (Figures 4D, 5D), pERK (Figures 4E, 5E), pJNK (Figures 4F, 5F), pp38 (Figures 4G, 5G), pAkt (Figures 4H, 5H), pmTOR (Figures 4I, 5I), pCREB (Figures 4J, 5J), pNFκB (Figures 4K, 5K), Nav1.7 (Figures 4L, 5L), Nav1.8 (Figures 4M, 5M), Iba1 (Figure 4N, 5N), S100B (Figure 4O, 5O), and RAGE (Figures 4P, 5P) following CIP induction. Also in accord with findings in the DRG, these changes were attenuated by AI and lower in CIP model *Trpv1*^−/−^ mice than in WT mice.

**Figure 6:**
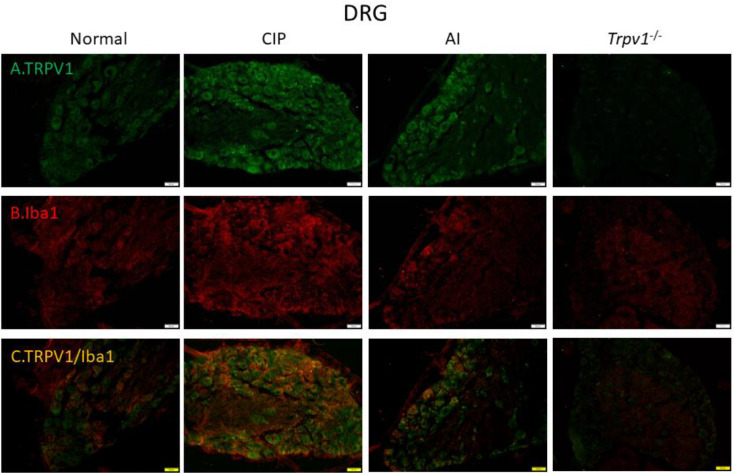
Immunofluorescence staining of transient receptor potential V1 (TRPV1) and Iba1 protein expression in the mice dorsal root ganglion (DRG). There are 4 groups: Normal, chronic inflammatory pain (CIP), acupoint injection (AI), and *Trpv1*^-/-^. (A) The efficacy of CIP treatment involves a significant increase of TRPV1 (green) in the mice DRG. (B) Significant increase of Iba1 (red) of CIP treatment in the mice DRG. (C) Overexpression of co-localization of TRPV1 and Iba1 (yellow) in the mice DRG. The scale bar is 50 μm

**Figure 7:**
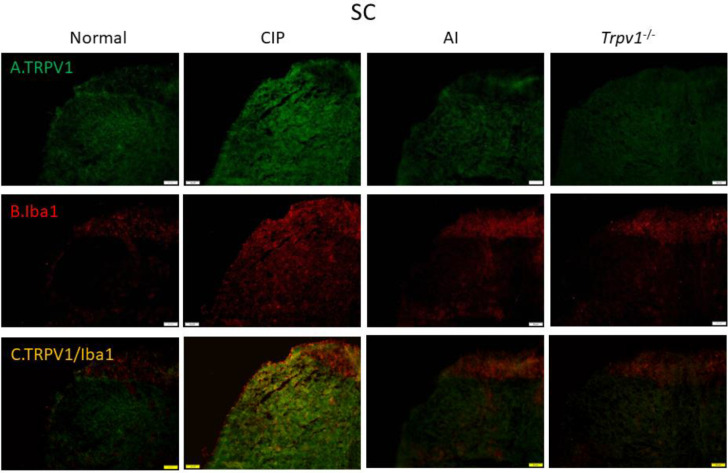
Immunofluorescence staining of Transient receptor potential V1 (TRPV1) and Iba1 protein expression in mice spinal cord (SC). There are 4 groups: Normal, chronic inflammatory pain (CIP), acupoint injection (AI), and *Trpv1*^-/-^. (A) The efficacy of CIP treatment involves significant increase of TRPV1 (green) in mice DRG. (B) Significant increase of Iba1 (red) of CIP treatment in mice DRG. (C) Overexpression of co-localization of TRPV1 and Iba1 (yellow) in mice DRG. The scale bar is 50 μm

To examine the cellular elements responsible for these expression changes, we conducted immunofluorescence staining of the DRG (Figure 6) and somatosensory cortex (Figure 7). Immunofluorescence images of the DRG revealed changes in TRPV1 immunoexpression that paralleled those measured by western blotting, with elevation following CIP induction compared with sham control mice and markedly lower expression levels in AI and *Trpv1*^-/-^ groups (Figure 6A). Immunofluorescence staining also confirmed elevated expression of the glial cell marker Iba1 in the DRG following CIP induction, which was attenuated by AI treatment and *Trpv1* gene deletion (Figure 6B). Further, TRPV1 and Iba1 were colocalized in CIP model mice, indicating that the elevation in tissue TRPV1 was mediated by increased expression in DRG glial cells. Consistent with western blotting results, this increase was reversed in AI and *Trpv1*^-/-^ mice (Figure 6C). Similar changes in TRPV1 and Iba1 expression were also observed in the somatosensory cortex (Figure 7).

***Co-expression of TRPV1 and Iba1 in cells of the thalamusand somatosensory cortex***

**Figure 8:**
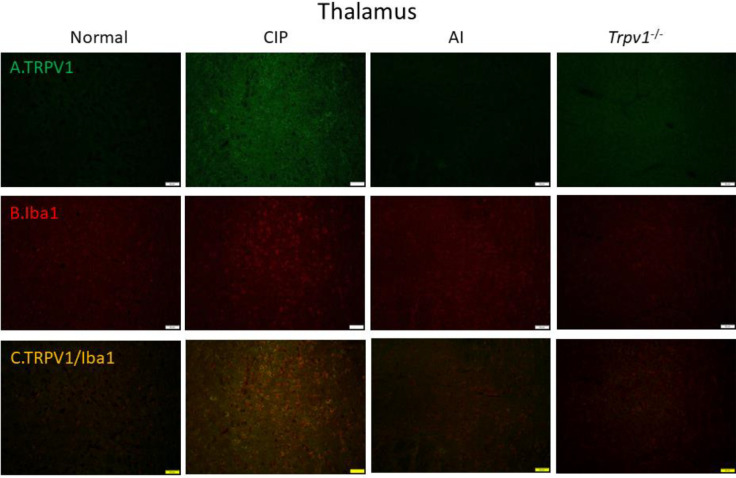
Immunofluorescence staining of transient receptor potential V1 (TRPV1) and Iba1 protein expression in the mice thalamus. There are 4 groups: Normal, chronic inflammatory pain (CIP), acupoint injection (AI), and *Trpv1*^-/-^. (A) The efficacy of CIP treatment involves significant increase of TRPV1 (green) in mice dorsal root ganglion (DRG). (B) Significant increase of Iba1 (red) of CIP treatment in mice DRG. (C) Overexpression of co-localization of TRPV1 and Iba1 (yellow) in mice DRG. The scale bar is 50 μm

**Figure 9:**
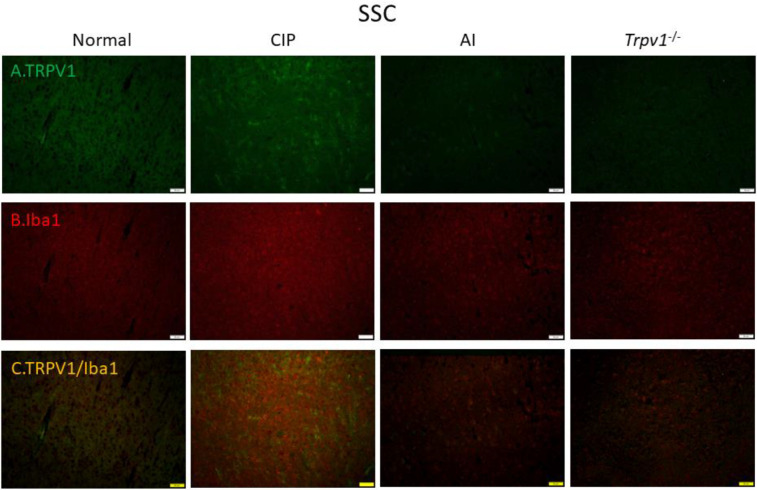
Immunofluorescence staining of transient receptor potential V1 (TRPV1) and Iba1 protein expression in the mice somatosensory cortex (SSC). There are 4 groups: Normal, chronic inflammatory pain (CIP), acupoint injection (AI), and *Trpv1*^-/-^. (A) The efficacy of CIP treatment involves significant increase of TRPV1 (green) in mice DRG. (B) Significant increase of Iba1 (red) of CIP treatment in mice DRG. (C) Overexpression of co-localization of TRPV1 and Iba1 (yellow) in mice DRG. The scale bar is 50 μm

Finally, we examined these changes in cellular TRPV1 and Iba1 in the thalamus (Figure 8) and somatosensory cortex (Figure 9). In accord with western blotting and other immunofluorescence results, TRPV1 immunoreactivity (Figure 8A) and Iba1 immunoreactivity (Figure 8B) were elevated concomitantly by CIP induction. Further, these elevated expression levels were reversed by AI (Figures 8A and 8B, respectively), while Iba1 expression was lower in CIP model *Trpv1*^-/- ^mice than WT mice (Figure 8C). In the somatosensory cortex as well, TRPV1, Iba1, and TRPV1/Iba1 co-staining signals were all increased in CIP model mice and reversed by AI (Figure 9). Further, the changes in Iba1 were suppressed in *Trpv1*^−/−^ mice.

(A) Mechanical hyperalgesia measured by von Frey test in the four groups. (B) Thermal hyperalgesia was assessed by the Hargreaves test in the four groups. Normal saline injection (Normal group, n = 8), CIP (CFA-induced CIP), AI (CFA-induced CIP with AI), and *Trpv1*^-/- ^(CFA-induced CIP in *Trpv1*^-/- ^mice). *P<0.05 vs the Normal group

CIP: chronic inflammatory pain; CFA: complete Freund’s adjuvant

## Discussion

In the present study, we present evidence that the hyperalgesia induced by CFA and the analgesic efficacy of AI are dependent on reciprocal modulation of TRPV1 channels expressed by glial cells in the DRG, SC, thalamus, and somatosensory cortex. Two weeks after CFA injection, mice demonstrated both mechanical hyperalgesia as evidenced by increased von Frey filament sensitivity, and thermal hyperalgesia as evidenced by Hargreaves’ thermal response test. These changes were associated with up-regulation of TRPV1 channels, a myriad of downstream kinases and transcription factors, and voltage-gated sodium channels (Na_v_s). Conversely, both forms of hyperalgesia were suppressed by AI treatment which was accompanied by reduced expression of TRPV1, associated signaling molecules, and Na_v_s. Further, these hyperalgesia-associated increases in downstream signaling molecules and Na_v_s were markedly suppressed in *Trpv1*^-/-^ mice. Expression levels of the activated microglial markers Iba1 and S100B were also up-regulated by CIP induction, and these responses were reversed by AI. Collectively, these findings indicate that TRPV1 signaling in microglia is a major driver of CFA-induced hyperalgesia and a primary therapeutic target of AI.

TRPV1 is also implicated in ST36 acupoint treatment, suggesting crucial involvement in the neurobiology of chronic pain and clinical potential as a treatment target (24). The influx of cations, particularly Ca^2+^, through TRPV1 channels activates the PI3K/Akt/mTOR and MAPK signaling pathways (25-27), and simultaneous overexpression of PKA, PI3K, PKC, ERK, Akt, and mTOR has been observed in various chronic pain states, while reversal of overexpression has been observed after successful acupuncture (28). Among transcription factors implicated in clinical pain conditions, CREB and pNFκB appear to mediate the gene expression changes observed in CIP model mice. Overexpression of the voltage-gated sodium channels Nav1.7 and Nav1.8 have also been observed in the DRG and SC of acute pain model animals (29). Conversely, suppression of Nav1.7 was associated with long-lasting analgesia (30). Furthermore, Nav1.8 is highly expressed by nociceptive sensory afferents and expression increases during chronic pain (31). The present study provides evidence that AI of glucose alleviates mechanical and thermal hyperalgesia by suppressing this increase in Nav1.8 through down-regulation of TRPV1 signaling in both PNS and CNS glial cells.

A recent study by He *et al*. (2017) provided evidence that nociceptive neuropeptides such as SP, CGRP, and 5-HT are highly expressed at acupoints (32), implicating modulation of these signaling pathways in AI-induced analgesia. Huang *et al*. (2018) also suggested that mast cells are crucial components of acupoints and can be activated by TRPV2 channels during acupuncture. A randomized, double-blinded, placebo-controlled clinical trial found that AI of onabotulinum toxin A (BoNTA) was more effective at suppressing pain than muscle injection in rats. They further suggested that BoNTA may suppress migraine through changes in CGRP and SP release within the medulla oblongata, possibly via a SNAP-dependent mechanism (33). Other researchers also reported that acupoint gel embedding could significantly diminish myocardial infarction size and inflammatory responses via the Notch-1 signaling pathway (34). Our previous publication indicated the detailed molecular mechanisms triggered by EA to attenuate inflammatory pain through the TRPV1 signaling pathway and related molecules. We suggest that EA can inhibit the release of S100B and consequently S100B binding to the RAGE receptor. We also recommended that EA can inhibit TRPV1 and reduce the downstream molecules associated with the expression of Nav1.7 and Nav1.8. EA may trigger endomorphin and adenosine release to decrease inflammatory pain (35). Our results extend the potential mechanisms underlying the benefits of AI to include analgesia via modulation of glial TRPV1 expression and signaling.

## Conclusion

In summary, the present study indicates that CFA can reliably induce CIP via up-regulation of TRPV1 signaling and that AI can suppress CIP by reversing this up-regulation. In addition, the activated microglial markers Iba1 and S100B demonstrated similar expression changes and were colocalized with TRPV1. These findings suggest that AI can mitigate chronic pain by suppressing TRPV1 overexpression in both neuronal and microglial cells.

## Authors’ Contributions

HYL and MCL Provided concept, methodology, data curation, visualization, investigation, and wrote the original draft. YLW Supervised, validated, wrote, reviewed, and edited the manuscript.

## Conflicts of Interest

The authors declare that no conflict of interest exists.
